# Obliterative bronchiolitis associated with rheumatoid arthritis: analysis of a single-center case series

**DOI:** 10.1186/s12890-018-0673-x

**Published:** 2018-06-22

**Authors:** Erica Lin, Andrew H. Limper, Teng Moua

**Affiliations:** 1 Department of Internal Medicine, 200 First St. SW, Rochester, MN 55905 USA; 2 Division of Pulmonary and Critical Care Medicine, Mayo Clinic, 200 First St. SW, Rochester, MN 55905 USA

**Keywords:** Rheumatoid arthritis, Obliterative bronchiolitis, Small airways disease

## Abstract

**Background:**

Rheumatoid arthritis (RA) is a systemic autoimmune condition characterized by erosive inflammation of the joints. One rare pulmonary manifestation is obliterative bronchiolitis (OB), a small airways disease characterized by the destruction of bronchiolar epithelium and airflow obstruction.

**Methods:**

We retrospectively reviewed the clinical data of patients with rheumatoid arthritis-associated obliterative bronchiolitis (RA-OB) from 01/01/2000 to 12/31/2015. Presenting clinical features, longitudinal pulmonary function testing, radiologic findings, and independent predictors of all-cause mortality were assessed.

**Results:**

Forty one patients fulfilled criteria for diagnosis of RA-OB. There was notable female predominance (92.7%) with a mean age of 57 ± 15 years. Dyspnea was the most common presenting clinical symptom. Median FEV1 was 40% (IQR 31–52.5) at presentation, with a mean decline of − 1.5% over a follow-up period of thirty-three months. Associated radiologic findings included mosaic attenuation and pulmonary nodules. A majority of patients (78%) received directed therapy including long-acting inhalers, systemic corticosteroids or other immunosuppressive agents, and macrolide antibiotics. All-cause mortality was 27% over a median follow-up of sixty-two months (IQR 32–113). No distinguishable predictors of survival at presentation were found.

**Conclusions:**

RA-OB appears to have a stable clinical course in the majority of patients despite persistent symptoms and severe obstruction based on presenting FEV1.

## Background

Rheumatoid arthritis (RA) is a systemic autoimmune condition characterized primarily by erosive inflammation of the joints affecting approximately 1% of the world’s population [1]. It is often associated with high morbidity due to extra-articular manifestations. Pulmonary manifestations of rheumatoid arthritis include airways disease, interstitial lung disease, parenchymal nodules, pleural disease, and pulmonary vasculitis [1–3]. These pulmonary findings can present early in the disease course [4] and account for up to 10% of rheumatoid arthritis-associated deaths [5, 6].

Obliterative bronchiolitis (OB, synonymous with constrictive bronchiolitis) is a rare small airways disease characterized by the destruction of bronchiolar epithelium and subsequent progressive airflow obstruction [7]. It has been found in a wide range of clinical settings. While obliterative bronchiolitis after transplantation has been well-described [8, 9], its presentation in non-transplant patients is still poorly understood and continues to be a source of interest [10]. Outside of organ transplantation, obliterative bronchiolitis is most commonly associated with connective tissue diseases, such as rheumatoid arthritis [8, 9]. Unfortunately, due to its rarity, there is still a paucity of literature regarding the clinical course of rheumatoid arthritis-associated obliterative bronchiolitis (RA-OB). It has been previously described as variable, in that some patients have periods of prolonged stability while others have a more rapid decline with respiratory failure and death [7]. Therefore, its longitudinal progression should be further evaluated for appropriate management and counseling of patients.

The majority of published studies represent individual case reports or case series, often characterizing the suspected association with penicillamine therapy used for management of RA [11–17]. One concern in assessing the functional history of such patients is the contribution of FEV1 decline from smoking. In a large case series that described both rheumatoid arthritis-associated follicular and obliterative bronchiolitis in fifteen biopsy-proven cases, a third had history of tobacco use [18]. The largest published retrospective study on RA-OB to date included over half with radiologic evidence of emphysema, perhaps confounding the severity of pulmonary function testing [19]. The purpose of this study was to characterize the clinical course and overall prognosis of patients with suspected RA-OB in the absence of radiologic emphysema.

## Methods

### Study population

Institutional Review Board approval was obtained (IRB #16–009418). The electronic medical record was searched to identify patients with RA-OB using key terms “rheumatoid arthritis” and “bronchiolitis.” Consecutive patients with suspected RA-OB evaluated at our institution from 1/1/2000 to 12/31/2015 were reviewed. Rheumatoid arthritis was defined according to criteria established by the American College of Rheumatology [20], confirmed with formal Rheumatology consultation. Obliterative bronchiolitis was defined using the following criteria: 1) presence of active compatible respiratory symptoms including dyspnea or cough plus 2) abnormal pattern on pulmonary function testing (PFT) in the absence of radiologic emphysema or 3) evidence of small airways disease on high-resolution computed tomography (CT) of the chest such as mosaicism or air trapping or centrilobular nodules or 4) histopathologic features on lung biopsy consistent with OB, when available in 5) the absence of an alternative diagnosis that could account for the findings. Indeed, there is not a standardized definition of RA-OB. The above-mentioned definition is based off findings by Hertz et al. [8]. Final diagnoses were made by multidisciplinary review at the time of clinical presentation. In order to select patients with obstructive airways disease attributed to suspected OB alone, patients were excluded if there was concomitant emphysema on imaging. Presenting clinical features, longitudinal PFTs, radiologic findings, therapeutic interventions, and all-cause mortality were collected from the electronic medical record. PFTs were performed in our laboratory using Medical Graphics equipment through standardized protocols by an experienced technician [21]. Collated PFT findings included percent predicted total lung capacity (TLC%), forced vital capacity (FVC%), forced expiratory volume in the first second (FEV1%), and diffusing capacity for carbon monoxide (DLCO%). Airflow obstruction was defined as FEV1 to FVC ratio less than the lower limit of normal (LLN), with staging of obstructive disease based on GOLD criteria for FEV1 [22]. Bronchodilator response was defined as spirometric findings of FEV1 improvement greater than 12% and g200 mL from baseline spirometry. High-resolution CT images were obtained using a scanning protocol in which 10–20 mm images were reconstructed using a high-spatial-resolution algorithm. Along with air-trapping or mosaicism, radiologist assessed presence of other rheumatoid-related pulmonary processes such as suspected rheumatoid lung nodules, lung fibrosis or fibrotic disease, pleural effusion, and non-traction related bronchiectasis, were collated. Biopsies were obtained by either surgical or transbronchial approach and reviewed by an experienced thoracic pathologist at the time of biopsy.

### Statistical analysis

Statistical analysis was performed using JMP Software (Version 10.0, SAS Corporation 2012). Quantitative variables were expressed as mean ± standard deviation or median and interquartile range, and qualitative variables as counts and percentage. Comparison of PFT at diagnosis and last available follow-up was assessed using Wilcoxon signed-rank test. Independent predictors of all-cause mortality were assessed using univariable Cox proportional hazards regression. Analysis was performed with statistical significance defined as two-tailed *p* values < 0.05.

## Results

Eighty-nine patients were found using the aforementioned key search terms. Upon review of the cases, forty-eight were excluded: eighteen had obliterative bronchiolitis secondary to other etiologies, seventeen had an incomplete diagnosis of obliterative bronchiolitis not meeting study criteria, eight did not have obliterative bronchiolitis, three were excluded for radiologic emphysema, and two did not have rheumatoid arthritis. After exclusion of non-qualifying patients, forty-one fulfilled the criteria for diagnosis of RA-OB. In most patients, the clinical, physiologic and radiologic findings precluded the need for confirmatory histopathologic diagnosis.

Demographic characteristics are presented in Table 1. There was notable female predominance (93%F:7%M) with a mean age of 57 ± 15 years (range, 22 to 84) at the time of diagnosis. Six were smokers without radiologic evidence of emphysema. Patients had a diagnosis of RA on average fourteen years before the diagnosis of RA-OB. Four patients were diagnosed with small airways disease either concomitantly or during the same year of their RA diagnosis. One patient had an overlap syndrome with RA and systemic lupus erythematosus (SLE). During the study period, patients received a wide range of therapeutic agents. Ten percent were previously on D-penicillamine. Twenty-four percent had non-pulmonary extra-articular manifestations. Specifically, 17% had secondary Sjögren’s syndrome. In addition to obliterative bronchiolitis, the majority had other rheumatoid arthritis-associated pulmonary disease, most commonly pulmonary nodules and/or bronchiectasis.

**Table 1:** Demographics and Clinical Presentation (*N* = 41)

Age at diagnosis (mean ± SD)	57 ± 15
Sex	
Male, N (%)	3 (7.3)
Female, N (%)	38 (92.7)
Positive tobacco use, N (%)	6 (14.6)
Pack year history (median (IQR))	13.5 (6.1-37.5)
Duration of RA prior to diagnosis, years (mean ± SD)	14 ± 12
Use of penicillamine, N (%)	4 (9.8)
Use of gold salts, N (%)	8 (19.5)
Non-pulmonary, extra-articular symptoms at diagnosis, N (%)	10 (24.4)
Active joint symptoms at diagnosis, N (%)	16 (39.0)
Sjögren’s syndrome	7(17)
Respiratory symptoms at diagnosis	
Dyspnea, N (%)	38 (92.7)
Cough, N (%)	13 (31.7)
Chest Pain, N (%)	8 (19.5)
Radiologic findings of OB at diagnosis (N=39)	
Mosaic attenuation, N (%)	20 (51.3)
Centrilobular nodules, N (%)	3 (7.7)
Radiologic findings of other RA-related pulmonary disease (N=39)	
Pulmonary nodules, N (%)	21 (53.8)
Fibrosis, N (%)	12 (30.8)
Pleural effusion, N (%)	2 (5.1)
Bronchiectasis, N (%)	19 (48.7)
Patients biopsied, N (%) (*one patient had both wedge and transbronchial*)	11 (26.8)
Wedge biopsy, N (%)	5 (41.7)
Transbronchial biopsy, N (%)	7 (58.3)
Unique biopsies consistent with OB diagnosis, N (%)	7 (58.3) (5 wedge, 2 TBB)

Abbreviations: *IQR* interquartile range, *RA* rheumatoid arthritis, *OB* obliterate bronchiolitis, *TBB* transbronchial biopsy

Dyspnea was the primary respiratory symptom, present in nearly all patients (92.7%). Thirty-nine percent had active joint symptoms at the time of diagnosis. Eleven underwent histologic assessment, consisting of surgical or transbronchial biopsy. Fifty percent of histopathologic slides were confirmatory, the rest were non-diagnostic with the majority showing non-specific chronic inflammation. Interestingly, all five surgical biopsies resulted in confirmatory histopathologic findings. In comparison, only two of the seven transbronchial biopsies resulted in confirmatory findings. This included one patient who underwent both wedge and transbronchial biopsies. Chest CT was obtained in 95% of patients, which revealed mosaic attenuation in 51.3%. Expiratory views were performed in 72% of CT studies. Physiologic studies were obtained at diagnosis and at last available follow-up (Table 2). Eighty-five percent had evidence of obstruction at initial assessment. There appeared to be no statistically significant absolute change in TLC%, FEV1%, FVC%, and DLCO% obtained at diagnosis and last available follow-up (median duration between PFTs of 33 months (IQR 14.6–51.7)). Mean percent change per year was also calculated in those with available follow-up study for each PFT parameter. Mean FEV1% change per year was − 0.4% with a mean increase in TLC% of 1.5% per year.

**Table 2:** Pulmonary Function Testing (PFTs)

	PFTs at diagnosis (*N* = 40)	Last PFTs (*N* = 25)	Total mean change from first to last PFT, % predicted (N = 25)	Mean % change per year	*P*-value^¶^
Obstruction, N (%)	34 (85.0)	21 (84.0)			
TLC% (median (IQR))	106 (88.0–115.0)	112 (105.0–123.0)	4.8	1.5 (*N* = 15)	0.46
RV/TLC% (median (IQR))	158 (144.0–181.5)	152 (146.3–166.8)	− 6.6	− 1.7 (*N* = 14)	0.07
FVC% (median (IQR))	66 (53.0–75.0)	61 (54.5–74.5)	− 1.9	− 0.5 (N = 25)	0.42
FEV1% (median (IQR))	40 (31.0–52.5)	41 (29.5–48.0)	−1.5	−0.4 (*N* = 25)	0.43
DLCO% (median (IQR))	69 (57.5–78.0)	64 (57.0–76.8)	−2.3	−0.6 (*N* = 18)	0.34
Duration between first and last available PFT, months (median (IQR) and mean (SD))	33(14.6–51.7), 42 (40)				

Abbreviations: *TLC%* percent of predicted total lung capacity, *RV/TLC%* percent of predicted residual volume over total lung capacity, *FVC%* percent of predicted forced vital capacity, *FEV1%* percent of predicted forced expiratory volume in the first second

¶Wilcoxon signed rank test for comparing two related samples

Thirty-two patients received directed medical therapy for RA-OB including long-acting inhalers (bronchodilators and inhaled corticosteroids), systemic corticosteroids or other immunosuppressive agents, and macrolide antibiotics (Table 3). Five patients were on supplemental oxygenation, and one underwent lung transplantation. Patients were followed for median duration of 61.7 months (IQR 32.1–113.1). All-cause mortality was 27% over the follow-up timeline. Clinical variables were assessed for their contribution to all-cause mortality (Table 4). No distinguishable predictors of survival at presentation were found on Cox regression analysis.

**Table 3:** Treatment and Outcome

Directed therapy (*N* = 32, 78.0%)	
Long-acting inhaler, N (%)	27 (84.4)
Systemic corticosteroids, N (%)	10 (31.3)
Macrolides, N (%)	18 (56.3)
Oxygen supplementation at rest	
Less than or equal to 3 L O2, N (%)	4 (12.5)
Greater than 3 L, N (%)	1 (3.1)
Transplantation, N (%)	1 (3.1)
Mortality, N (%)	11 (26.8)
Time from OB diagnosis to death, months (mean ± SD)	54 ± 53

**Table 4:** Univariable Cox regression predictors of all-cause mortality

Characteristics	HR (95% CI)	*P*-value
Age at diagnosis, years	0.99 (0.94–1.05)	0.78
Gender, female	0.93 (0.15–17.6)	0.94
Duration of RA prior to diagnosis, years	0.98 (0.94–1.03)	0.58
Active joint symptoms at diagnosis	1.87 (0.24–11.42)	0.51
Mosaic attenuation at diagnosis	0.59 (0.12–2.17)	0.44
Lung fibrosis at diagnosis	2.12 (0.58–7.76)	0.24
Bronchiectasis at diagnosis	1.02 (0.28–3.69)	0.97
Baseline PFTs at diagnosis		
TLC%	0.99 (0.95–1.04)	0.76
RV/TLC%	1.01 (0.98–1.03)	0.51
FVC%	0.94 (0.87–1.03)	0.19
FEV1%	0.99 (0.91–1.07)	0.96
Positive bronchodilator response	3.41 (0.73–17.7)	0.11
Macrolide therapy	1.73 (0.40–7.44)	0.44

Abbreviations: *TLC%* percent of predicted total lung capacity, *RV/TLC%* percent of predicted residual volume over total lung capacity, *FVC%* percent of predicted forced vital capacity, *FEV1%* percent of predicted forced expiratory volume in the first second, *RA* rheumatoid arthritis

## Discussion

To our knowledge, this study represents the largest case series to date of patients with rheumatoid arthritis-associated obliterative bronchiolitis. The syndrome occurs primarily in middle-aged women after more than a decade of rheumatoid arthritis. The majority presented with dyspnea and radiologic findings of mosaic attenuation and centrilobular nodules. Serial pulmonary function testing demonstrated severe (median FEV1 40%) but stable obstructive disease over time. Seventy-eight percent received directed therapy including long-acting inhalers, systemic corticosteroids or other immunosuppressive agents, and macrolide antibiotics. All-cause mortality was 27% over a median follow-up of five years, without any distinguishable predictors of survival at presentation on Cox regression analysis.

Obliterative bronchiolitis has been described in the literature as a manifestation of underlying rheumatologic disease or secondary to its therapeutic agents. Geddes et al. first described several cases in 1977 [11]. During their initial reports, the syndrome appeared to be more prevalent in patients on D-penicillamine therapy, raising the possibility of drug-induced bronchiolopathy [12, 13, 16]. However, patients treated with D-penicillamine for other conditions, such as Wilson’s disease, did not develop similar airway findings [12, 16]. In our study, only 10% were previously on D-penicillamine.

The majority of our patients were female. This has also been observed in other studies [16, 23] and may reflect the female predominance associated with rheumatoid arthritis. Interestingly, Fernandez Perez et al. noted that male gender was an independent predictor of mortality in connective tissue disease-associated obliterative bronchiolitis [24]. Our study did not show a similar relationship between gender and mortality.

The mean interval between diagnosis of rheumatoid arthritis and obliterative bronchiolitis was fourteen years. This duration is longer than the five to ten years found in other studies [18, 19]. The diagnosis of RA-OB may be delayed in patients due to lack of recognition by local providers and thus reflect referral bias in a tertiary care center. It may also be confounded by earlier diagnosis of rheumatoid arthritis. Similar to other studies [16, 18, 19], we found that initial clinical presentation of airways disease was non-specific with predominant symptoms of dyspnea and cough. Less than 40% had active joint symptoms at diagnosis, similar to findings by Devoussoux et al. [19]. A quarter of patients had non-pulmonary and extra-articular manifestations, most commonly secondary Sjögren’s. Geli et al. reviewed the CT findings of patients with primary Sjögren’s and noted that 65% had bronchiolar abnormalities [25]. This incidence may be a reflection of the association between bronchiolopathies and generalized connective tissue disorders. It is unclear whether secondary Sjögren’s affects the development or clinical course of obliterative bronchiolitis in patients with RA. Further studies to evaluate the relationship between obliterative bronchiolitis and primary versus secondary Sjögren’s syndrome are needed.

While our study did not evaluate chest radiographs, prior studies reported radiologic findings such as hyperinflation and hyperlucency [18, 19]. We found that half of our patients had characteristic findings of mosaic attenuation with expiratory air trapping on CT [18]. We suspect the frequency of mosaic attenuation may be under-estimated as expiratory images were not routinely obtained on chest CT at presentation(only 72% had specific expiratory images). We note that Devouassoux et al. found a high prevalence of emphysema on CT in patients with RA-OB, even in nine non-smokers [19]. We specifically excluded those with radiologic evidence of emphysema given concern for confounding of PFT severity and long-term survival by this finding. This may have resulted in a more biased subset of individuals, not reflecting the possible co-existence of small airways disease and emphysema in non-smokers. In general, patients with rheumatoid arthritis often have several or multiple radiologic abnormalities on high-resolution chest CT. Remy-Jardin and colleagues found the most common abnormalities were bronchiectasis and bronchiolectasis [26], as airways disease is often the earliest manifestation of RA in the lung [3]. In contrast, Tanaka et al. reported a higher prevalence of interstitial findings, such as ground glass opacities or reticulation. This discrepancy may be the result of referral bias, as many of the patients included in this study were first evaluated in an interstitial lung disease clinic [27]. In our series, the most common radiologic finding was pulmonary nodules, followed by bronchiolar abnormalities. The predominance of bronchiectasis is consistent with RA with or without obliterative bronchiolitis. In advanced cases of obliterative bronchiolitis, there may be wall thickening in both small and large airways. Interestingly, only 30% of our patients had evidence of fibrosis. This lower percentage may be a reflection of gender differences, as rheumatoid arthritis-associated pulmonary fibrosis is more common in males than females [3]. None of these additional findings on their own contributed to an increased risk of mortality, as assessed by univariate Cox regression analysis, likely due to the limited sample size and number of events (deaths) in our series.

Diagnosis of obliterative bronchiolitis may be confirmed with biopsy, either surgical or transbronchial. In our study, all surgical biopsies resulted in confirmatory histopathologic findings, compared to only two of seven transbronchial biopsies. This included one patient with both wedge and transbronchial biopsies. In our small sample size, the diagnostic yield of surgical biopsy was superior to transbronchial biopsy. Further studies to better understand the diagnostic yield of each modality are needed. While the diagnosis of RA-OB may be obtained clinically, in patients with suspected but indeterminate findings requiring histopathologic confirmation, we recommend surgical biopsy for greater yield, if feasible and safe.

In our study, pulmonary function tests were typically consistent with an obstructive pattern. There was a minority of patients with non-specific, restrictive, or normal findings. Diffusing capacity was relatively maintained in comparison to other airway or parenchymal diseases such as chronic obstructive pulmonary disease (COPD) or interstitial lung disease (ILD) associated with RA. While other studies have noted that FEV1 was significantly lower in patients previously on D-penicillamine [19], we did not find evidence of worsening pulmonary impairment in patients on this agent. Fernandez Perez et al. investigated the longitudinal progression of pulmonary function testing in patients with connective tissue disease associated-obliterative bronchiolitis [24]. While their study did not distinguish between underlying rheumatoid arthritis versus other connective tissue diseases, they discovered that the rate of FEV1 change was minimal during their three-year follow-up, regardless of therapy [24]. Similarly, our study cohort had minimal change in FEV1, suggesting the clinical and functional course of RA-OB may stabilize for a period of time in some.

Given the paucity of literature regarding this syndrome, current evidence for therapy is limited. Case reports mention corticosteroids [16] and other immunosuppressive agents including azathioprine [14], cyclophosphamide [23], and etanercept [28] as possible therapeutic agents directed at small airways disease. Case series have demonstrated that macrolides improve or slow the progression of respiratory impairment in RA-OB [18], which is also evident for other bronchiolar diseases [29]. Unfortunately, large randomized trials for RA-OB are lacking due to disease rarity. Our observational data reveal that medical therapy often included a combination of long-acting inhalers, systemic corticosteroids or other immunosuppressive agents, and macrolide antibiotics. Despite these options, treatment of obliterative bronchiolitis does not appear to reverse disease. It is unclear if FEV1 stabilization in our cohort was a result of natural clinical course or directed therapy. We found that all-cause mortality was 27% over a median follow-up of five years. While the severity of disease based on clinical symptoms and functional findings on PFTs was poor, there again appeared to be a fairly stable clinical course in the majority with gradual deterioration on a time scale of years. This is in contrast to other studies that highlight obliterative bronchiolitis as severe or advancing with immediate decline over months [11–14]. It remains unclear why there is such heterogeneity in disease course.

Our study has several limitations. This is a single center study assessing patients diagnosed and managed over a number of years, reflecting local practices and their evolution over time. Patients referred to tertiary care centers may have more advanced or refractory disease, and thus may not be fully representative of the general disease population. Patients also had physiologic studies measured at varied intervals over time which make accurate assessment of rates of decline less reliable over the study interval. Because of the lack of standardized therapy, we were unable to make inferences about the efficacy of any particular treatment regimen on pulmonary function testing or clinical course. Lastly, as a retrospective study, exact circumstances involved in decision making are difficult to determine, particularly causal effects of management. Despite being one of the larger retrospective studies to date, the absolute number of patients still limited statistical analyses, including multivariable comparisons.

## Conclusions

Rheumatoid arthritis-associated obliterative bronchiolitis appears to have a fairly stable clinical course despite persistent symptoms and severe airflow obstruction. While diagnosis may be obtained clinically using supporting radiographic evidence, in patients with suspected disease requiring histopathologic confirmation, we recommend performing surgical biopsy for greater yield, if feasible and safe. Interestingly, serial pulmonary function testing showed only mild deterioration in FEV1 over time. There were no distinguishable predictors at presentation to frame the likelihood of therapeutic response or more rapid clinical decline. Characterizing this longitudinal progression though has valuable implication in the appropriate counseling of patients and future design of clinical trials.

## Abbreviations

COPD: Chronic obstructive pulmonary disease
CT: Computed tomography
DLCO: Diffusing capacity for carbon monoxide
FEV1: Forced expiratory volume in the first second
FVC: Forced vital capacity
ILD: Interstitial lung disease
IQR: Interquartile range
LLN: Lower limits of normal
OB: Obliterative bronchiolitis
PFT: Pulmonary function testing
RA: Rheumatoid arthritis
RA-OB: Rheumatoid arthritis-associated obliterative bronchiolitis
RV/TLC: Residual volume over total lung capacity
SLE: Systemic lupus erythematosus
TBB: Transbronchial biopsy
TLC: Total lung capacity
